# Comparison of translucency, thickness, and gap width of thermoformed and 3D-printed clear aligners using micro-CT and spectrophotometer

**DOI:** 10.1038/s41598-023-36851-5

**Published:** 2023-07-05

**Authors:** So Yeon Park, Sung-Hwan Choi, Hyung-Seog Yu, Su-Jung Kim, Hoon Kim, Ki Beom Kim, Jung-Yul Cha

**Affiliations:** 1 Department of Orthodontics, College of Dentistry, Yonsei University, Seoul, South Korea; 2 Department of Orthodontics, Institute of Craniofacial Deformity, College of Dentistry, Institute for Innovation in Digital Healthcare, Yonsei University, 50-1 Yonsei-ro, Seodaemun-gu, Seoul, 03722 South Korea; 3 Department of Orthodontics, Kyung Hee University School of Dentistry, Seoul, South Korea; 4 Laboratory of Adhesion & Bio-Composites, Forestry and Bioresources, Seoul National University, Seoul, South Korea; 5 Department of Orthodontics, Saint Louis University, Saint Louis, MO USA

**Keywords:** Medical research, Materials science

## Abstract

The present study compared the thickness and gap width of thermoformed and 3D-printed clear aligners (CAs) using micro-computed tomography (micro-CT) and evaluated their translucency using spectrophotometer. Four groups of CAs were tested: thermoformed with polyethylene terephthalate glycol (TS) or copolyester-elastomer combination (TM), and 3D**-**printed TC-85 cleaned with alcohol (PA) or with centrifuge (PC). CIELab coordinates were measured (n = 10) to evaluate translucency. CAs (n = 10) were fitted onto respective models and micro-CT was performed to evaluate the thickness and gap width. Thickness and gap width were measured for different tooth type and location in sagittal sections on all sides. The PC group showed significantly higher translucency than the PA group, which was similar to the TS and TM groups (*p* < 0.01). After the manufacturing process, thickness reduction was observed in the thermoformed groups, whereas thickness increase was observed in the 3D printed-groups. The TM group showed the least gap width amongst the groups (*p* < 0.01). Thermoformed and 3D-printed CAs had significantly varied thicknesses and regions of best fit depending on the tooth type and location. Differences in the translucency and thickness of the 3D-printed CAs were observed depending on the cleaning methods.

## Introduction

Clear aligners (CAs) need to exert a continuous and controlled force on teeth to achieve the desired tooth movement^1^. They are produced from a variety of thermoplastic materials, including polyethylene terephthalate glycol (PETG), thermoplastic polyurethane (TPU), polypropylene, and polycarbonate^1,2^. PETG is a commonly preferred material for CAs because of its higher translucency, fatigue resistance, and dimensional stability^2^. TPU is a ductile elastomer with high elasticity and formability, which provides good wearability and shock absorption^3,4^. In addition, multi-layer hybrid materials have been introduced to overcome the drawbacks of single-layer materials. Materials with a combination of hard outer shells and a soft inner shell show improved mechanical strength of maximum load in the tensile test and water absorption rate^5^.

The clinical performance of CAs has been enhanced by development new materials with good mechanical properties. However, the conventional fabrication process, which involves vacuum thermoforming of thermoplastic materials, is both time- and labor-intensive for producing serial models and trimming the aligners along the gingival margin at each stage^6,7^. This process also has adverse environmental effects, such as plastic waste and energy consumption^8^. In addition, the thermoforming process may result in unpredictable changes in physical properties of the CAs, making it difficult to predict its treatment outcomes^6,7^. Varying degrees of shrinkage and expansion are observed in thermoplastic materials after the thermoforming process^9^, which can affect the thickness and fit of aligners. A previous study that evaluated the thickness and gap width of CAs after the manufacturing process found variations in these parameters for all thermoplastic materials depending on tooth type and location, such as the incisor, canine, and molar teeth or buccal and palatal sides^10^.

The thickness and gap width of CAs are clinically important factors for increasing the predictability of their performance^11^. The thickness of CAs can affect the forces and moments needed for tooth movement^11–13^. The close fit between tooth surfaces and CAs is an crucial factor in allowing effective force to be transferred to the teeth and plays an effective role in anchorage^10,14^. As patients seeking orthodontic treatment often prioritize esthetic features, including color stability and translucency, the translucency is also considered a significant clinical factor^15,16^.

With the development of three dimensional (3D) printers and their materials, CAs have begun to be integrated with digital technologies. Advances in technology allow clinicians to take intraoral scans, set up the virtual tooth movement, and directly produce CAs using a 3D printer without taking impressions or using plaster models^17^, minimizing the cumulative errors from the thermoplastic workflows^6^. In addition, 3D printing is associated with shorter manufacturing time and high efficiency when producing a series of aligners simultaneously^18^, lower costs and efforts, and less waste material and environmental pollution^17,19^. As a result, the application of CAs has expanded, and their fabrication reproducibility has improved^17^.

Currently, several research methodologies for evaluating the accuracy of CAs are being studied. Cole et al. found that the dimensional accuracy of thermoformed and 3D-printed CAs evaluated using scanning and superimposition techniques with geometric software at specific tooth landmarks ranged from 0.1 to 0.3 mm and from 0.1 to 0.4 mm, respectively^20^. Jindal et al. also found that the geometrical accuracy of thermoformed and 3D-printed CAs, in terms of tooth height, ranged between 0–0.88 mm and 0.02–0.86 mm, respectively^6^. Although these results were concluded to be clinically acceptable, the dimensional accuracy of CAs shows a wide range. In addition, spray application is required to scan the highly reflective or transparent surface of the CAs with the above method, which may affect accuracy^21^. Meanwhile, comparing the dimensional accuracy of the aligners fitted to the models using micro computed tomography (micro-CT) scanning is advantageous because the technique is non-invasive and precise^10,22^. Lombardo et al. attempted to evaluate the thickness and gap width of aligners using cone-beam computed tomography^10^. However, their study was limited to examining only thermoformed materials and performing repeated measurements of a single sample.

Furthermore, the post-processing step is crucial to enhance the printing accuracy of the 3D-printed CAs^23,24^. 3D-printed CAs require post-processing immediately after printing^25^, and this typically involves three steps: support removal, cleaning, and post-curing^26^. Different cleaning methods to remove any uncured resin residue from 3D-printed CAs are recommended by manufacturers depending on the material, and it can also have an impact on the surface roughness and mechanical properties of the manufactured aligners^23,24^. The most commonly used solvent to remove excess uncured resin from aligners is isopropyl alcohol (IPA), which effectively dissolves esters^27^. Nevertheless, IPA should be used as a solvent with caution due to its high volatility and flammability, that can lead to respiratory problems with prolonged exposure^28^.

Recently, resin materials for 3D-printed CAs with good mechanical, optical, and biocompatible properties have been developed^29^. The development of photo-polymerizable polyurethane (Tera Harz TC-85, Graphy Inc., Seoul, Korea) has received approval from the Korea and United States Food and Drug Administration for biocompatibility^30^. TC-85 has viscous and flexible characteristics, which can continuously apply light forces on the teeth and exhibits creep behavior^31^. Additionally, it has shape-memory properties and dimensional stability at high temperatures^31^. The manufacturing company of TC-85 recommends a non-chemical cleaning method called centrifugation as an alternative to IPA^32^. However, the effect of different cleaning methods on the translucency, thickness, and gap width in the post-processing of the TC-85 3D-printed CAs has not yet been tested.

Therefore, the present study evaluated the null hypothesis that different fabrication protocols have no significant effects on the translucency, thickness, and gap width of CAs. The specific objectives of this study were to evaluate the micro-level variations in thickness and gap width between different tooth types and locations.

## Results

### Comparison of translucency for CAs using different fabrication protocols

The translucency values were significantly higher in the TS, TM, and PC groups than in the PA group (*p* < 0.01) (Table 1).

**Table 1:** Median translucency parameter for CAs using different fabrication protocols.

	Median (IQR)				*p*-value	Post-hoc
	TS (n = 10)	TM (n = 10)	PA (n = 10)	PC (n = 10)		
Translucency parameter	67.82 (67.64–68.05)	66.15 (65.84–66.45)	10.76 (9.17–15.81)	66.19 (65.44–66.36)	0.001*	TS,PC,TM > PA

*p*-values were calculated using Kruskal–Wallis test for multiple comparisons, followed by post hoc comparisons using Mann–Whitney U test with a Bonferroni adjustment of alpha level. *p <  0.01.

### Comparison of thickness and gap width for CAs using different fabrication protocols

There were significant differences in thickness and gap width among the four groups (*p* < 0.01). The median thicknesses of the TS, TM, PA, and PC groups were 504.68 μm, 509.54 μm, 614.24 μm, and 687.53 μm, respectively. The median gap widths of the TS, TM, PA, and PC groups were 69.80 μm, 52.35 μm, 69.80 μm, and 69.80 μm, respectively. The median thickness was significantly higher in the PC group compared to the other groups (*p* < 0.01). The median gap width was significantly higher in the PA, PC groups than in the TM group (*p* < 0.01) (Table 2).

**Table 2:** Median thickness and gap width for CAs using different fabrication protocols.

	Median (IQR) (μm)				*p*-value	Post-hoc
	TS (n = 10)	TM (n = 10)	PA (n = 10)	PC (n = 10)		
Thickness	504.68 (460.68–558.40)	509.54 (467.66–548.80)	614.24 (559.27–687.53)	687.53 (639.54–749.48)	0.001*	PC > PA > TS,TM
Gap width	69.80 (0–115.17)	52.35 (0–89.87)	69.80 (17.45–107.32)	69.80 (34.90–131.75)	0.001*	PA,PC > TM

*p*-values were calculated using Kruskal–Wallis test, followed by post hoc comparisons using Mann–Whitney U test with a Bonferroni adjustment of alpha level. *p < 0.01. There was no significant difference in gap width between the TS and TM group.

### Group comparisons for thickness depending on tooth type and location

In the TS, PA, and PC groups, the median thickness was greater for the anterior teeth than for the posterior teeth (TS: *p* < 0.05; PA and PC: *p* < 0.01).

**Figure 1:**
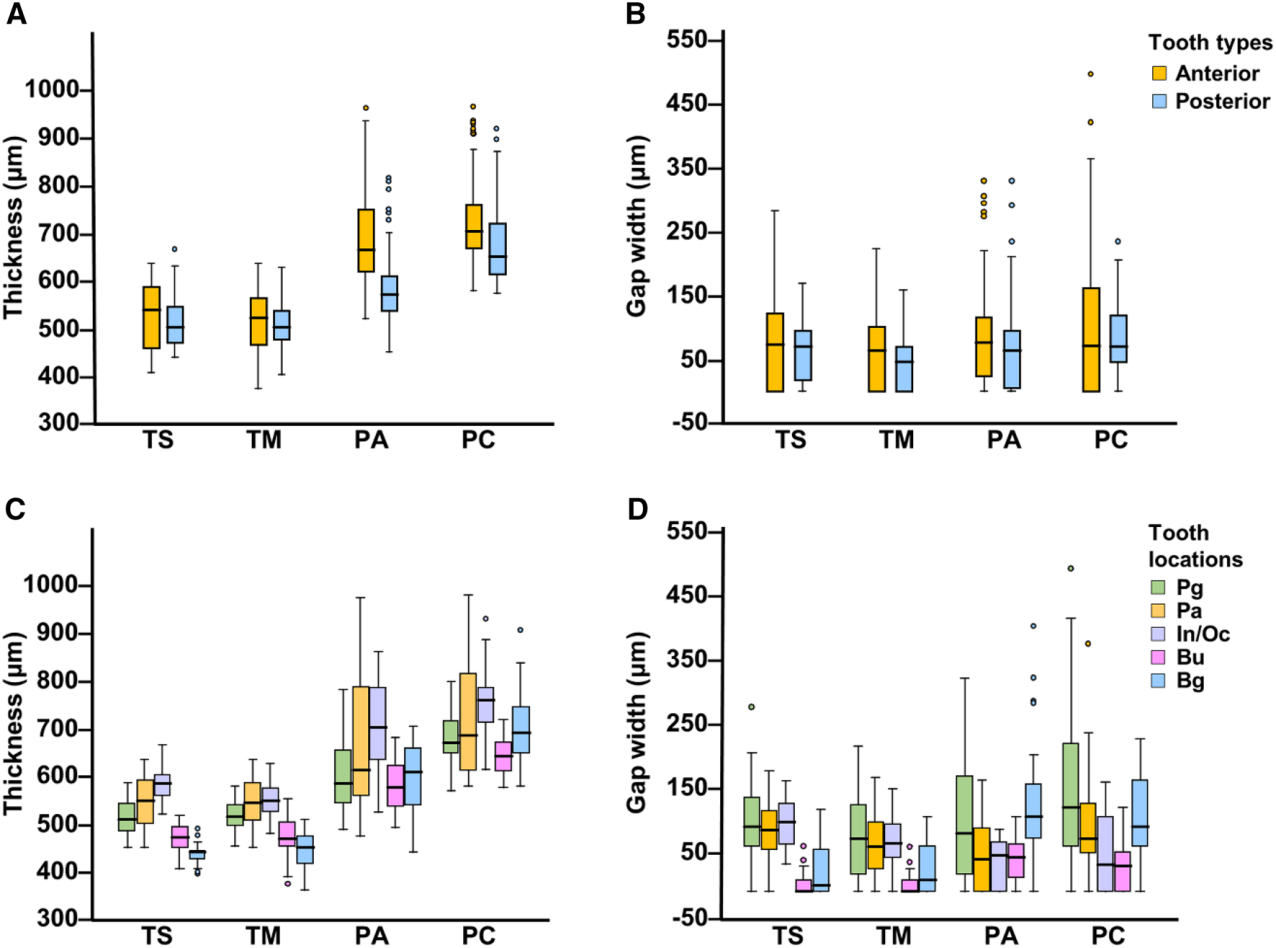
Median (IQR) thickness and gap width for four clear aligners according to tooth type and location. (**A**) and (**C**), median thickness by tooth type and location; (**B**) and (**D**), median gap width by tooth type and location. Outlier (1.5 × IQR) values are shown by closed circles. *Pg*: palatogingival; *Pa*: palatal; *In/Oc*: incisal or occlusal; *Bu*: buccal; *Bg*: buccogingival.

Group variations in the median thickness were observed as follows: TS: buccogingival < buccal < palatogingival < palatal < incisal or occlusal; TM: buccogingival < buccal < palatogingival < palatal, incisal or occlusal; and PA, PC: buccal, palatogingival, buccogingival < palatal < incisal or occlusal. The thickness variations are further detailed in Table 3 and Fig. 1A,C.

**Table 3:** Group comparisons for median thickness depending on tooth type and location.

Tooth type/tooth location	Median (IQR) (μm)			
	TS (n = 10)	TM (n = 10)	PA (n = 10)	PC (n = 10)
Anterior teeth	537.46 (451.08–588.94)	518.27 (460.68–563.64)	668.34 (621.22–759.08)	710.22 (670–95-770.42)
Posterior teeth	499.07 (464.17–544.44)	509.54 (467.66–548.80)	568.87 (533.97–613.37)	654.38 (615.11–727.67)
*p*-value	0.021*	0.639	0.001**	0.001**
Post-hoc	Anterior > posterior	–	Anterior > posterior	
Palatogingival	513.03 (488.60–547.06)	516.52 (499.07–543.57)	588.07 (547.93–660.48)	673.57 (652.63–721.56)
Palatal	551.42 (502.56–595.92)	547.93 (505.18–591.56)	615.99 (560.15–797.47)	689.28 (615.11–822.77)
Incisal/occlusal	588.07 (560.43–606.97)	550.26 (527.86–579.34)	706.15 (636.35–793.10)	763.15 (716.32–789.61)
Buccal	474.64 (453.70–498.20)	471.15 (457.19–506.05)	579.34 (538.33–629.07)	645.65 (614.24–676.19)
Buccogingival	443.23 (429.27–445.85)	453.70 (419.67–478.13)	610.75 (541.82–663.10)	694.51 (652.63–749.48)
*p*-value	0.001**	0.001**	0.001**	0.001**
Post-hoc	*Bg* < *Bu* < *Pg* < *Pa* < *In/Oc*	*Bg* < *Bu* < *Pg* < *Pa,In/Oc*	*Bu,Pg,Bg* < *Pa* < *In/Oc*	

*p*-values were calculated using Mann–Whitney U test for comparisons by tooth type.

*p*-values were calculated using Kruskal–Wallis test for multiple comparisons by tooth location, followed by post hoc comparisons using Mann–Whitney U test with a Bonferroni adjustment of alpha level. *p < 0.05; **p < 0.01.

### Group comparisons for gap width depending on tooth type and location

In the TM group, the median gap width was greater for the anterior teeth than for the posterior teeth (*p* < 0.05), while the median gap width for the anterior and posterior teeth were similar in the other groups.

**Figure 2:**
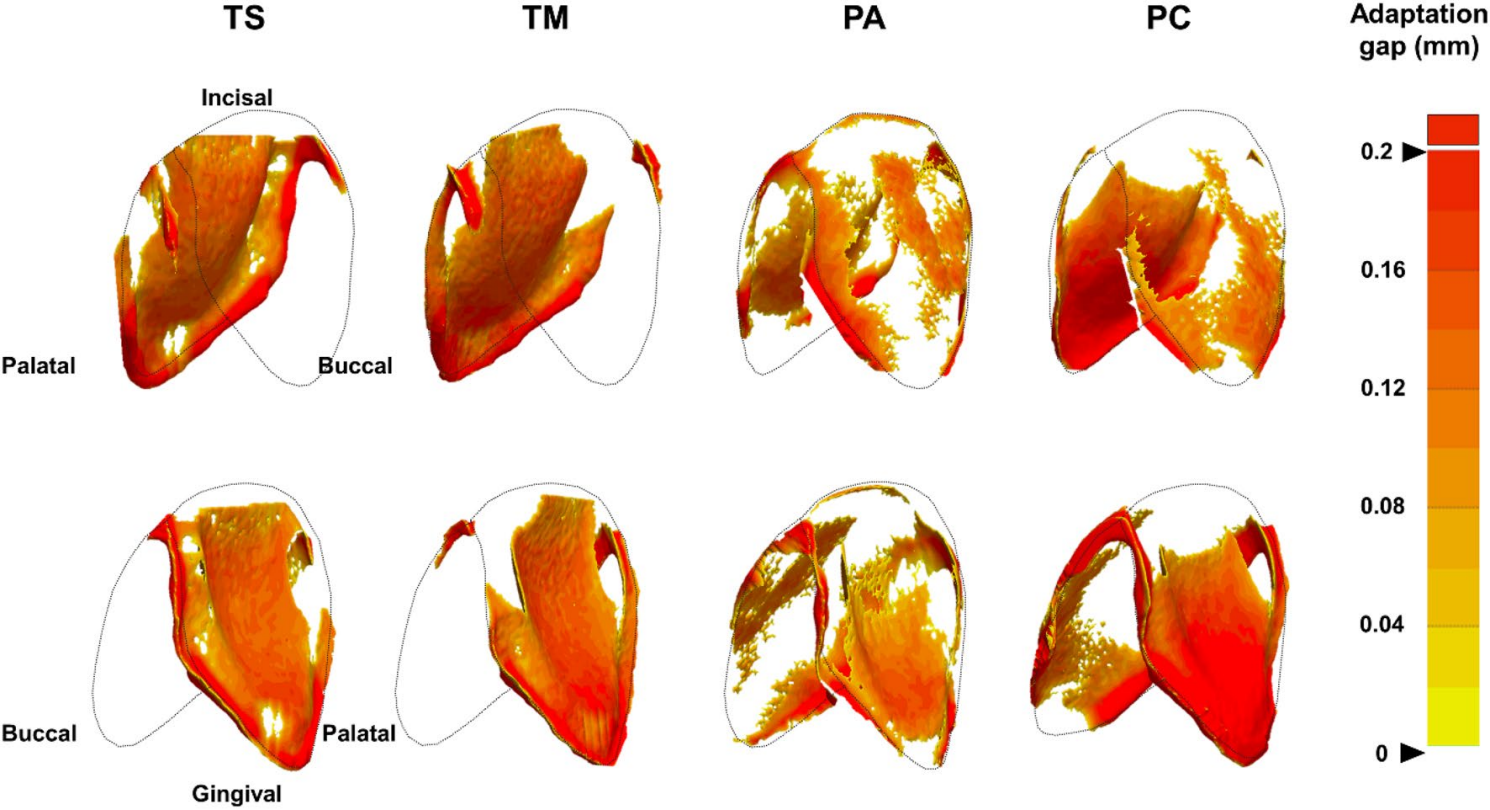
Representative images of the three-dimensional distribution of the gap along the central incisor with different group of clear aligners. The color scale represents the amount of the gap from 0 to 200 µm (yellow to red). The blank (empty) region within the tooth outline represents areas with negligible gap.

Group variations in the median gap width were observed as follows: TS: buccal, buccogingival < palatal, palatogingival, incisal or occlusal; TM: buccal, buccogingival < palatal, incisal or occlusal, palatogingival; PA: palatal < buccal, incisal or occlusal < palatogingival, buccogingival; and PC: buccal, incisal or occlusal < palatal, buccogingival, palatogingival. The gap width variations are further detailed in Table 4 and Fig. 1B,D. The four clear aligner groups showed differences in 3D-distribution of the gap along the central incisor (Fig. 2).

**Table 4:** Group comparisons for median gap width depending on tooth type and location.

Tooth type/tooth location	Median (IQR) (µm)			
	TS (n = 10)	TM (n = 10)	PA (n = 10)	PC (n = 10)
Anterior teeth	73.29 (0–122.15)	62.82 (0–102.96)	76.78 (24.43–115.17)	71.55 (0–162.29)
Posterior teeth	69.80 (17.45–95.98)	45.37 (0–69.80)	63.41 (2.62–94.23)	69.80 (45.37–118.37)
*p*-value	0.677	0.045*	0.079	0.083
Post-hoc	–	Anterior > posterior	–	–
Palatogingival	99.47 (69.80–145.71)	80.27 (21.81–134.37)	89.00 (21.81–189.33)	129.13 (69.80–233.83)
Palatal	94.23 (61.95–124.77)	68.06 (34.90–107.32)	48.86 (0–99.47)	80.27 (59.33–135.24)
Incisal/occlusal	105.87 (73.29–135.82)	74.46 (52.35–105.86)	55.84 (0–76.78)	40.72 (0–114.88)
Buccal	0 (0–17.45)	0 (0–17.45)	52.35 (19.20–75.04)	38.39 (0–61.95)
Buccogingival	8.73 (0–67.18)	17.45 (0–69.8)	115.17 (81.14–164.03)	99.47 (69.80–172.76)
*p*-value	0.001**	0.001**	0.001**	0.001**
Post-hoc	*Bu,Bg* < *Pa,Pg,In/Oc*	*Bu,Bg* < *Pa,In/Oc,Pg*	*Pa* < *Bu,In/Oc* < *Pg,Bg*	*Bu,In/Oc* < *Pa,Bg,Pg*

*p*-values were calculated using Mann–Whitney U test for comparisons by tooth type.

*p*-values were calculated using Kruskal–Wallis test for multiple comparisons by tooth location, followed by post hoc comparisons using Mann–Whitney U test with a Bonferroni adjustment of alpha level. *p < 0.05; **p < 0.01.

## Discussion

Transparency is the physical property that allows light to pass through a material, which is an important factor in determining the esthetics of the CAs and is the major concern of individuals seeking orthodontic treatment^15,16^. The thickness and gap width of CAs are also important factors that can have the greatest influence on orthodontic treatment^10,12,14^. The thickness of CAs can be used as a predictive factor by orthodontist to control the physiological forces and moments applied to the teeth during treatment^33^. During orthodontic treatment, the physiological forces applied to the teeth enable resorptive remodeling and controlled tooth movement^34,35^. However, overloading the teeth with excessive forces can result in side effects, such as cell death, tissue hyalinization of the periodontal ligament, alveolar bone necrosis, and external root resorption^36^. The inner surface of the aligner must be as close to the teeth as possible to provide a clinically effective force^10^. Moreover, a reduced fit can cause aligners to lift up during the torquing process of root movements, making it difficult to establish an effective force couple^37^.

In addition, the post-processing procedure is a crucial step as it can affect the printing accuracy and mechanical properties of the printed objects^23,24^. However, there is insufficient evidence-based data to evaluate the changes in the translucency, thickness, and gap width of TC-85 3D-printed CAs according to different cleaning methods. Therefore, this study used IPA and centrifugation as the two chemical and non-chemical cleaning methods for evaluating the outcomes of the 3D-printed samples.

Thermoformed CAs made from thermoplastic materials could be worn immediately on the teeth. However, 3D-printed CAs developed using the TC-85 material required a clinically different wearing protocol, as recommended by the manufacturer due to their special properties, which was applied in this study. Based on the geometric stability at high temperatures and shape-memory property of TC-85, 3D-printed CAs were immersed in 80 °C water, which is higher than the glass transition temperature^31^. This immersion was performed to increase flexibility of the aligners before placing them on the models^31^. Afterward, the sample gradually recovered its original shape and fit when kept at a temperature of 37 °C, indicating that the deformation was reversible^31^. In summary, the softening of aligners by warm water provided the comfortable wear to the models. Thereafter, the samples were dried at a temperature of 37 °C to recover their original shape and strength.

There were differences in translucency among the CAs manufactured by different fabrication protocols. The PC group showed significantly higher translucency, similar to that of the TS and TM groups. On the other hand, the PA group showed a decrease in translucency. IPA is the most commonly used solvent for dissolving excess uncured resin^27^. However, previous studies have suggested that IPA, due to low compatibility with acrylate based polymer, tends to cause polymer swelling instead of dissolution^38,39^. Furthermore, as IPA evaporates from the swollen polymer surface, the polymer chains can undergo reorganization, resulting in changes in the surface’s solubility and leading to surface cracking of the 3D-printe objects^39^. Therefore, it could be inferred that IPA cleaning has a negative effect on the surface properties of the TC-85 3D-printed CAs.

Further, the findings of our study demonstrated that the thermoformed CAs decreased in thickness after the fabrication process, while the 3D-printed CAs increased in thickness compared to their set-up thickness. While an increase in overall thickness was observed in the PC group compared to the PA group, there was no significant difference in thickness after the thermoforming process of the TS and TM groups. In previous studies, it has been shown that thermoplastic materials experience a decrease in thickness during the thermoforming process, while 3D-printed aligners undergo an increase in thickness^13,31^. Lee et al. reported a 54.7% decrease in the thickness of PETG samples thermoformed on a standardized maxillary incisor model^31^. Edelmann et al. found that the 3D-printed CAs increase in thickness by approximately 0.2 mm after printing^21^.

Significant differences in thickness for each tooth type were observed among the CAs manufactured by different fabrication protocols. The TS group was less stretched at the anterior teeth than at the posterior teeth under heat and pressure. The PA and PC groups showed thicker output in the anterior teeth compared to the posterior teeth. The reason for the thickening of the anterior teeth in the 3D-printed groups is as follows: the anterior teeth are structurally tapered and have longer crowns. It is difficult to remove any uncured resin remaining on the inner surface before the final post-polymerization step^6^, which may cause an increase in thickness. When observing thickness variations according to tooth location, the TS and TM groups were thinner at the buccal and buccogingival areas. Thermoplastic materials showed a greater thickness reduction in the convex buccal surface and distant gingival areas as they wrapped and extended from the occlusal surface of the model. Further, the median thickness of the incisal/occlusal areas in the PA and PC groups was significantly greater than that in other locations. This appeared to be a printing error owing to the complex curvature of the tooth surface at the incisal edge, occlusal cusp tip, and occlusal central pit. This printing error can occur in the curved contour area during thickness layering, which is affected by the layer thickness, curvature radius, and inclination^40^.

In the present study, the TM group showed the least gap width amongst the groups. The multi-layer hybrid material consisted of copolyesters and a flexible elastomer core, which has better mechanical strength with a higher maximum load in the tensile test than a single-layer material^5^. Therefore, a multi-layer material might be more extensible during heating and pressure, resulting in a better fit. Different patterns of gap width depending on the tooth location between thermoformed and 3D-printed CAs were also observed. In the thermoformed groups, the incisal/occlusal surface showed the greatest thickness with less variation, but had the largest gap width. In the 3D-printed groups, the buccal side showed the least thickness and variation with a small gap width, whereas the incisal/occlusal surface demonstrated the greatest variation in thickness, resulting in the thickest aligner, but with the smallest gap width. However, the gingival areas on both sides had less thickness variation and showed the largest gap width in the 3D-printed CAs. This phenomenon occurs due to the accumulation of polymerization shrinkage during the printing process, that magnifies the unsuitability of the gingival margins^41^.

Based on previous studies, various parameter optimizations were performed to improve 3D printing accuracy in this study. Firstly, the post-curing process for 3D-printed CAs was performed under nitrogen conditions that prevented the formation of an oxygen inhibition layer, allowing the surface of the specimen to polymerize^42,43^. Injecting inert nitrogen gas to exclude oxygen can also help to achieve good mechanical properties and surface smoothness of the 3D-printed CAs^43^. Secondly, the build angle and layer thickness are particularly important settings for the accuracy of 3D printing. As a result, the 3D-printed CAs were designed at a build angle of 30° and printed using a digital light processing (DLP) 3D printer with a 50 µm resolution, based on previous studies^44,45^. Thirdly, a 50 µm offset was applied to the inner surface of the 3D-printed CAs to compensate for the thickness change and to enhance the adaptability^46^. A previous study examining the fit of a printed splint showed that splints with 0.05, 0.1, and 0.2 mm offsets can fit the teeth better than those without offset^46^. In addition, the dimensional accuracy of a 3D-printed aligners is affected by factors including light intensity, exposure time, and the properties of printing materials such as light-blocking pigment concentration and light penetration^44,47,48^. Therefore, it is important to calibrate printing parameters to enhance the printing accuracy and minimize variance in each output^49^.

Though the present study presented an objective assessment of differences among four CAs groups, the findings should be interpreted within the limitations. Although it is theoretically possible to set the thickness of 3D-printed CAs for comparison with thermoformed aligners, the recommendations for effective TC-85 3D-printed aligners have a lower limit of 0.5 mm. Therefore, the sample selection criteria were based on clinically recommended preferences. Taking the above into account, we selected a thickness of 0.75 mm (factoring in thickness reduction) for the thermoplastic materials and a thickness of 0.5 mm for our 3D-printed CAs, which is the optimal thickness currently applied in clinical practice. Even though the wearing protocol recommended by the manufacturer was followed to apply the 3D-printed CAs to the models, there were experimental constraints in creating an oral environment at 37 °C temperature with high relative humidity during micro-CT scanning, which may have affected the fit. The present study used only passive-state CAs with a normal occlusion model to obtain objective evaluation data on 3D-printed CAs, as there is no information on how 3D-printed CAs are manufactured and printed depending on tooth type and location. In the future, it will be important to observe differences in thickness and gap width when CAs are activated in various degrees of malocclusion, and to evaluate how attachments affect the thickness and gap width of the aligners. Furthermore, additional evaluation is needed to determine how the thickness and gap width observed in this study affect the clinical performance of achieving the desired tooth movement.

## Conclusion

Our null hypothesis that there is no difference in translucency, thickness, and gap width among groups manufactured by different fabrication protocols was rejected in this study. After the manufacturing process, the thermoformed CAs showed a reduction in thickness while the 3D-printed CAs showed an increase in thickness. The PA group showed the lowest translucency, and the TM group had the best fit amongst the groups. The thickness and regions of best fit of the thermoformed and 3D-printed CAs significantly varied depending on the tooth type and location. Additionally, the cleaning methods used in the post-processing of 3D-printed CAs affected their translucency and thickness.

## Materials and methods

### Sample preparation

A standardized model of the maxillary dental arch in Korean adults with normal occlusion (CON2001-UL-SP-FEM-32, Nissin Dental, Kyoto, Japan) was used and scanned using an intraoral scanner (D250, 3shape, Copenhagen, Denmark) to create an STL file. A standardized model of 60 mm × 50 mm × 20 mm was then printed (S-100, Graphy Inc., Seoul, Korea) using a DLP 3D printer (Asiga MAX™, Asiga, Alexandria, Australia).

Ten CA samples were prepared for each group. Thermoformed CAs were divided into a single-layer group (TS group) with a PETG sheet (Duran, Scheu-Dental, Iserlohn, Germany) and a multi-layer group (TM group) with copolyester in both outer shells and thermoplastic elastomer in the inner shell sheet (CA pro, Scheu-Dental, Iserlohn, Germany). According to the cleaning method, 3D-printed CAs with photo-polymerizable polyurethane resin (TC-85) were divided into two groups: a PA group with isopropyl alcohol cleaning and a PC group with centrifuge cleaning.

A 0.75-mm-thick thermoplastic material was vacuum-thermoformed on a standardized model using a thermomolding caster (Biostar, Scheu-Dental, Iserlohn, Germany) under the thermal deformation conditions recommended by the manufacturer. The thickness of the CAs may be affected by various conditions, such as model preparation, pressure, heat, and positioning of the model on the platform. Therefore, it is important to control these conditions as much as possible during manufacturing^22,50^. A uniform base was created by setting a constant height of the model from the teeth, and the model was placed at the center of the platform, such that the mid-palatal suture was oriented toward the 12 o'clock position. The orientation of the model was kept constant based on the markings for each thermoforming process. As the final step, the CAs were separated from the model and the gingival edge of the aligner was cut and polished.

**Figure 3:**
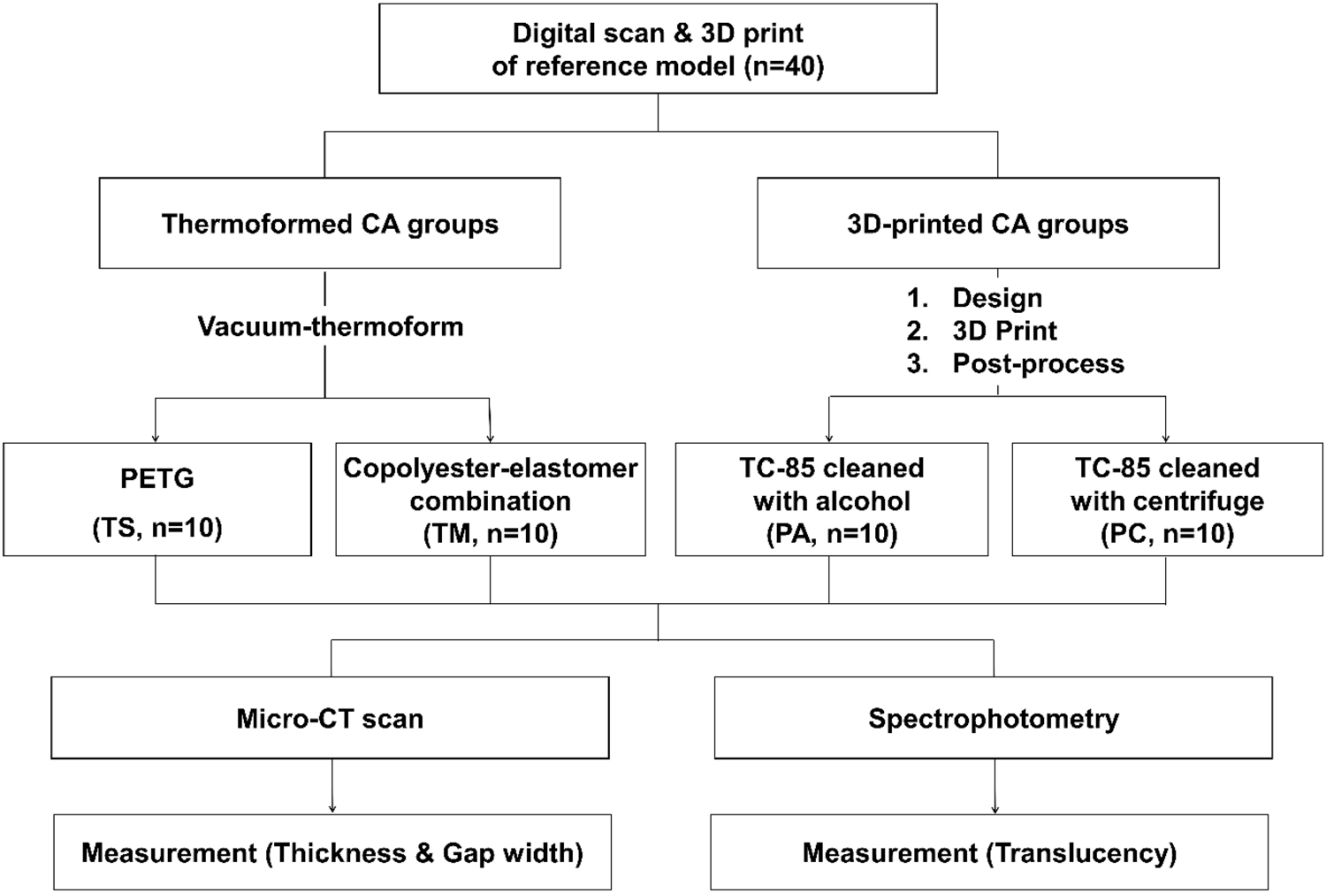
Flowchart of experimental design.

3D-printed CAs were designed using computer-assisted design software (Deltaface, Coruo, Limoges, France) with a set up thickness of 0.5 mm, offset of 50 μm, and positioned at 30° as a print angulation with minimum strut supports. A DLP 3D printer (SprintRay Pro 95, SprintRay, Los Angeles, CA) was used with a layer thickness of 50 μm. Two different cleaning methods were used to remove any uncured resin from the aligner surfaces. CAs were cleaned either for 1 min with 99.5% IPA or for 6 min and 500 rpm using a centrifuge. The samples were then cured twice for 25 min under nitrogen conditions with ultraviolet light (385–405 nm) using a post-curing chamber (CureM U102H, Graphy Inc., Seoul, Korea). Final cleaning was performed with flowing water and using an ultrasonic cleaner for 3 min at 76–80 °C. The diagram presented in Fig. 3 shows the experimental design.

### Thickness and gap width measurement method using micro-CT

**Figure 4:**
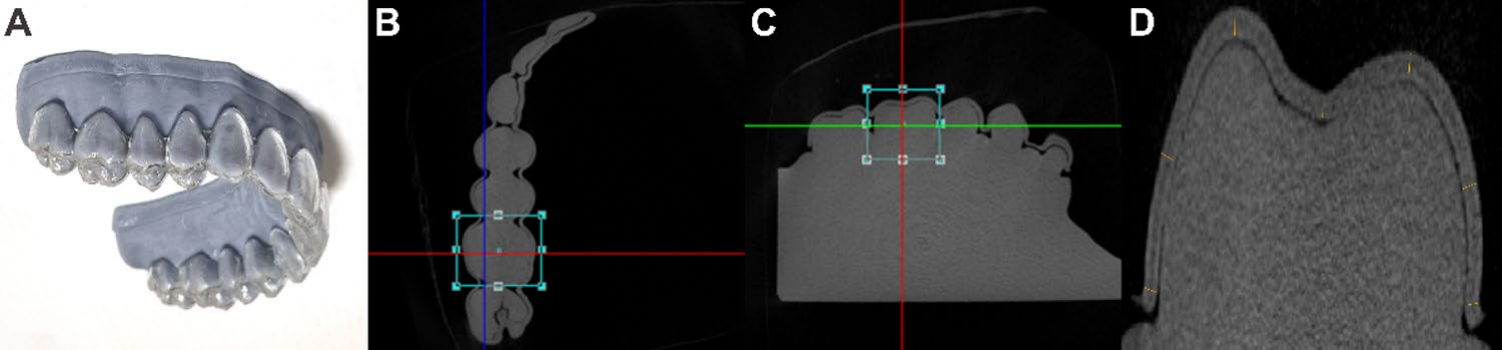
The measurement method of thickness and gap width using micro-CT. (**A**) A clear aligner was placed on a standardized model; (**B**) and (**C**) the slice plane setting of the target tooth obtained from micro-CT imaging and the application of VOI were performed using Dataviewer software; and (**D**) the thickness and gap width of the tooth were measured using CTAn software.

**Figure 5:**
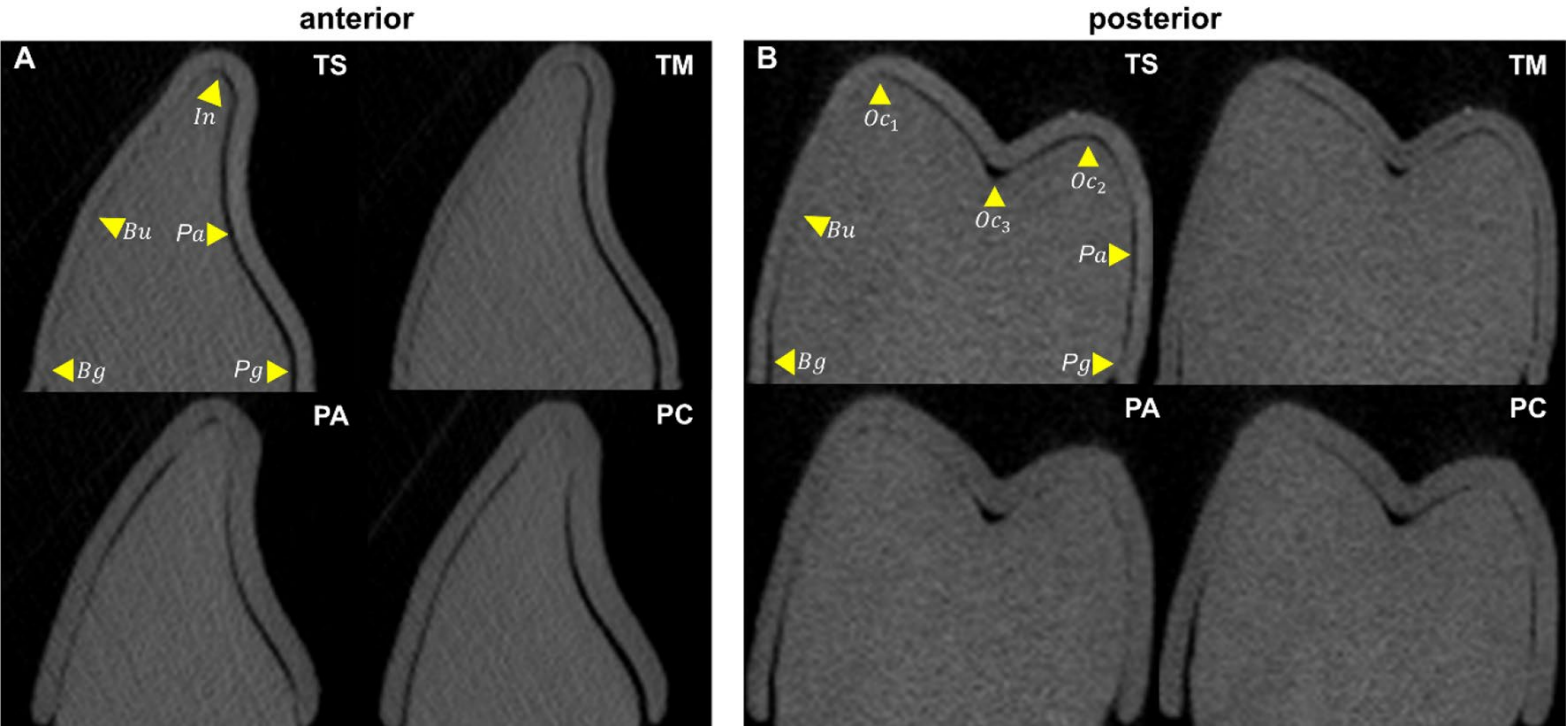
Microphotographs showing the thickness and gap width between aligners and teeth at reference points (tooth location). (**A**) anterior teeth (e.g., the central incisor); (**B**) posterior teeth (e.g., the first premolar). Yellow arrows show the reference points of teeth; *Pg,* palatogingival; *Pa,* palatal; *In/Oc,* incisal or occlusal; *Bu,* buccal; *Bg,* buccogingival.

The thermoformed CAs were immediately fitted onto the standardized model at room temperature, whereas the 3D-printed CAs were inserted after being smoothly transformed in warm water at 80 °C, following the manufacturer's clinical protocol. Afterwards, the 3D-printed samples were dried at 37 °C to restore their original shape and strength. All aligners (n = 10) were scanned using a high-resolution micro-CT (Skyscan1173, Bruker, MA, USA) at 40 kV, 200 μA, and 34.9 μm of resolution. A total of 40 micro-CTs were obtained, and the target areas of dentition (anterior teeth: the right maxillary central incisor, canine; posterior teeth: the right maxillary first premolar and molar) were re-orientated using Dataviewer software (version 1.5.6.2, Bruker, MA, USA). Slices were obtained using a horizontal plane from the model base and perpendicular to the middle of the line linking the most mesial and distal contact points of the tooth, and were saved by applying the volume of interest (VOI). The images were analyzed using CTAn software (release 2.5, Bruker, MA, USA) at 300× magnification (Fig. 4). Thereafter, the shortest distance of thickness and gap width were measured by projecting a perpendicular line from each reference point tangent. These 5–7 reference points were based on a pilot study and included gingival margins, buccal and palatal midpoints, and incisal/occlusal points (incisal edge, occlusal cusp tips, and central pit) (Fig. 5)^51^. The occlusal surface of the molars includes both the cusp tips and central pit structures, but they are clinically considered as a single plane. Therefore, instead of separating and analyzing their measurement values individually (${{Oc}_{1},{Oc}_{2},Oc}_{3}$), we interpreted them through mean values. A total of 960 points on tooth surfaces were included in the measurements, which were repeated twice at an interval of two weeks by one researcher.

### Translucency measurement method using spectrophotometer

Translucency is the difference in the color of a material with a uniform thickness over white and black backgrounds^52^. A spectrophotometer (CM-3500d, Konica Minolta, Tokyo, Japan) was used to calculate the CIELab coordinates of the specimens placed on white and black backgrounds. The window size of the spectrophotometer was 3 mm, and 10 samples with a 3 mm diameter of 0.75-mm-thick thermoformed and 0.5-mm-thick 3D-printed specimens were prepared. The following Eq. (1) was used to determine translucency:

1 $$\mathrm{Translucency\,parameter }=\sqrt{{\left({L}_{B}^{*}-{L}_{W}^{*}\right)}^{2}+{\left({a}_{B}^{*}-{a}_{W}^{*}\right)}^{2}+ {\left({b}_{B}^{*}-{b}_{W}^{*}\right)}^{2}}$$

Translucency parameter ranged from 0 (complete opacity) to 100 (complete transparency)^53^.

### 3D visualization of the gap

Using 3D Slicer software (version 5.0.3, http://www.slicer.org), the micro-CT Digital Imaging and Communications in Medicine (DICOM) files of the central incisors were analyzed. Manual segmentation was performed to obtain the 3D information of the gap between the tooth and the aligners. The manually segmented regions of interest were 3D-rendered and exported as STL files. The STL files representing the gap width between the tooth and aligners were morphometrically compared using Geomagic Control X software (version 2018.0.1, 3D Systems, SC, USA).

### Statistical analysis

SPSS software (version 25.0, IBM, NY, USA) was used for statistical analysis of the data. The Shapiro–Wilk test for normality was applied, and the normality of the distribution was rejected. A non-parametric Mann–Whitney U test and Kruskal–Wallis test were performed to compare the median translucency, thickness, and gap width of aligners depending on tooth type and location, followed by Mann–Whitney test with Bonferroni’s correction for multiple comparisons. The *p*-value for statistical significance (conventional level of 0.05) was divided by the number of statistical tests performed.

## Data Availability

All of the data supporting this work will be made available from the corresponding author upon reasonable request.
